# Diagnostic efficacy of a combination of the Chinese thyroid imaging reporting and data system and shear wave elastography in detecting category 4a and 4b thyroid nodules

**DOI:** 10.3389/fendo.2023.1161424

**Published:** 2023-06-12

**Authors:** Huizhan Li, Jiping Xue, Yanxia Zhang, Junwang Miao, Liwei Jing, Chunsong Kang

**Affiliations:** ^1^ Department of Ultrasonography, Shanxi Bethune Hospital, Shanxi Academy of Medical Sciences, Tongji Shanxi Hospital, Third Hospital of Shanxi Medical University, Taiyuan, Shanxi, China; ^2^ Department of Health Statistics, Shanxi Medical University, Taiyuan, Shanxi, China

**Keywords:** papillary thyroid carcinoma, shear wave elastography, thyroid, thyroid imaging reporting and data system, thyroid nodule, ultrasonography

## Abstract

**Objectives:**

Differential diagnosis of benign and malignant thyroid imaging reporting and data system (TIRADS) category 4a and 4b nodules can be difficult using conventional ultrasonography (US). The objective of this study was to evaluate the diagnostic efficacy of a combination of the Chinese-TIRADS (C-TIRADS) and shear wave elastography (SWE) in detecting malignant nodules among category 4a and 4b thyroid nodules.

**Methods:**

Among 409 thyroid nodules in 332 patients that we included in this study, 106 thyroid nodules were diagnosed as category 4a and 4b using C-TIRADS. We used SWE to measure the maximum Young’s modulus (Emax) values of category 4a and 4b thyroid nodules. We calculated the diagnostic efficacy of only the C-TIRADS, only SWE, and a combination of C-TIRADS with SWE, and compared these, while taking the pathology results as the gold standard.

**Results:**

The area under the ROC curve (AUC), sensitivity, and accuracy values of the combination of C-TIRADS and SWE (0.870, 83.3%, and 84.0%, respectively) were all higher when compared with the values of only the C-TIRADS (0.785, 68.5%, and 78.3%, respectively) or only SWE (0.775, 68.5%, and 77.4%, respectively) in the diagnosis of category 4a and 4b thyroid nodules.

**Conclusion:**

In this study, we found that the combination of C-TIRADS and SWE significantly improved the diagnostic efficacy in detecting malignant nodules among category 4a and 4b thyroid nodules, and this could provide a reference for further use of this combination by clinicians for diagnosis and treatment.

## Introduction

Thyroid nodules are very common worldwide, and are detected in approximately 19%–68% of the general population. The majority of these thyroid nodules are benign (1, 2). Ultrasound is the best imaging method for the diagnosis of thyroid nodules. However, it is still difficult to diagnose atypical benign and malignant nodules, which are often classified as category 4a or 4b as per the Thyroid Imaging Reporting and Data System (TIRADS) (3, 4). Category 4a and 4b thyroid nodules usually need to be referred for fine needle aspiration (FNA) biopsy to rule out or confirm malignancy. However, a wide range of malignancy rates for these nodules (3.3%–72.4%) is reported in literature (5). It is, therefore, necessary to identify complementary investigations that can improve the diagnostic efficacy of detecting category 4a and 4b thyroid nodules.

Shear wave elastography (SWE) is used to quantitatively measure tissue stiffness based on Young’s modulus values. The maximum value measured using SWE (Emax) is the most commonly used parameter, and it is used extensively in the diagnosis of benign and malignant thyroid nodules. The sensitivity and specificity of SWE for differentiating benign from malignant thyroid nodules are 0.79–0.86 and 0.84–0.90, respectively (6).

The purpose of this study was to evaluate the diagnostic efficacy of using a combination of conventional ultrasonography (US) and SWE to differentiate between benign and malignant category 4a and 4b thyroid nodules. The findings of this research have implications for improving the diagnostic efficacy of detecting such nodules and their clinical management. Considering that there are many different versions of thyroid ultrasound classification systems to diagnose benign and malignant thyroid nodules (7), we chose the Chinese Thyroid Imaging Reporting and Data System (C-TIRADS), which was recently released and more practical given the current status of medical treatment (8).

## Materials and methods

### Ethics declaration

This study was approved by the Medical Ethics Committee of the Shanxi Bethune Hospital(Taiyuan, Shanxi Province, China). This research was conducted in accordance with the relevant regulations and guidelines, and all participants or their legal guardians gave their signed written informed consent.

### Participants

In this study, we enrolled 332 consecutive patients (214 women and 118 men) with 409 thyroid nodules, who were treated in Shanxi Bethune Hospital (Taiyuan, Shanxi Province, China) between January 2019 to October 2021. Their median age was 45 years (range: 28–69 years).

The inclusion criteria were as follows: (1) Patients who underwent thyroid surgery and had positive pathology findings; (2) Patients with complete data, including US indicators and SWE data; and (3) Patients who had not been previously treated for thyroid nodules.

Among the total enrolled patients, 245 patients (73.8%) presented with a single nodule, and 87 patients (26.2%) had multiple nodules. The size of the 409 thyroid nodules ranged from 0.5–3.4 cm.

Histological findings after thyroid surgery were used as a reference for the diagnosis of malignant thyroid nodules.

### Ultrasonography examinations

Thyroid US and SWE examinations were performed with an Aixplorer US system (SuperSonic Imagine, Aix-en-Provence, France), which was equipped with an SL15-4 multifrequency linear array transducer. All nodules were examined by the same radiologist who was proficient in performing the SWE imaging procedure with more than 10 years of ultrasound work experience.

Patients were placed in the supine position with the neck fully exposed before the US examination began. As per the C-TIRADS, we assessed six features of each nodule, namely, internal structure, echogenicity, margin, calcification, aspect ratio, and comet-tail artifact. We assigned a corresponding score for each feature, and then the nodules were assigned different C-TIRADS classifications according to their total scores. Additionally, we measured the maximum diameter of each nodule.

SWE was performed with the same US machine and transducer after the US examination. The target nodule was displayed on the long-axis section of the thyroid, and the image was switched to SWE mode (display Young’s modulus scale: 0–100 kPa). A region of interest, including the whole target lesion and the surrounding normal thyroid tissue, was identified on the nodule, and the SWE image was captured and stored after stabilizing the image. Subsequently, the Emax value of the nodule was measured using the Q-box in three independent measurements, and the mean of the three Emax values was recorded for analysis.

### Scoring system

Two physicians with at least 5 years of ultrasound work experience independently evaluated all the ultrasonic images. In case of a disagreement, a third associate chief physician with more than 10 years of ultrasound work experience evaluated the image, it was discussed among the three physicians, and a consensus was reached.

We rated all thyroid nodules according to the C-TIRADS scoring system (8): Solid composition, microcalcifications, markedly hypoechoic, ill-defined or irregular margins, or extrathyroidal extensions, and vertical orientation were considered as malignant ultrasound features, while comet-tail artifacts were considered as indicating benign status. Risk stratification was calculated by adding the number of the above-mentioned malignant ultrasound features and then subtracting one (1) if negative features of the comet-tail artifacts were present.

TIRADS 1 (Score 0): no nodule;

TIRADS 2 (Score-1): benign nodules, including the so-called “white knight” nodules, which are referred to as uniform hyperechoic nodules that appear on a background of Hashimoto’s thyroiditis;

TIRADS 3 (Score 0): probably benign nodules, including nodular goiter;

TIRADS 4a (Score 1): low suspicious nodules (malignancy between 2% and 10%), including nodules with macrocalcifications or peripheral calcifications with strong acoustic shadowing;

TIRADS 4b (Score 2): moderately suspicious nodules (malignancy between 10% and 50%);

TIRADS 4c (Score 3, 4): highly suspicious nodules (malignancy between 50% and 90%);

TIRADS 5(Score 5): highly suggestive of malignancy(malignancy >90%), including nodules with a “snowstorm” pattern of microcalcifications;

TIRADS 6: biopsy-proven malignant nodules.

TIRADS 1 to TIRADS 4a were classified as benign, and TIRADS 4b to TIRADS 6 were classified as malignant.

In this study, 106 nodules were diagnosed as category 4a or 4b, which included 63 patients with category 4a nodules and 43 patients with category 4b nodules.

#### SWE classification standard

These nodules were also diagnosed using SWE, and the diagnostic criteria were based on our previous research results (9):

According to the size of the nodules, we used different cutoff points to diagnose the nodules.

Maximum diameter ≤ 1 cm: Emax ≥ 33.7 kPa, the nodule was diagnosed as malignant;

Maximum diameter 1–2 cm: Emax ≥ 37.7 kPa, the nodule was diagnosed as malignant;

Maximum diameter ≥2 cm: Emax ≥55.1 kPa, the nodule was diagnosed as malignant.

#### C-TIRADS + SWE classification standard

Then, we diagnosed the nodules using C-TIRADS + SWE:

If Emax ≥ cutoff points, nodules were regarded as having a higher TIRADS category.

### Statistical analysis

We used the R software package (R Foundation for Statistical Computing, Vienna, Austria) for all statistical analyses in our study. Two-tailed *P <*0.05 was considered to be statistically significant. We used the Shapiro–Wilk test for evaluating normality of the distribution. Descriptive statistics were expressed as medians (25^th^ and 75^th^ percentiles) or mean values ± standard deviations for continuous data. We assessed the diagnostic efficacy of each method in detecting category 4a and 4b thyroid nodules using the receiver operating characteristic (ROC) curve analysis. We calculated the area under the ROC curve (AUC), and the AUC values were compared using Z test. The accuracy, sensitivity, specificity, positive predictive value (PPV), and negative predictive value (NPV) were calculated. We used the McNemar test for comparison of sensitivity and specificity between the methods.

## Results

### Diagnostic efficacy of C-TIRADS in detecting malignant nodules among category 4a and 4b thyroid nodules

106 thyroid nodules were diagnosed using US as category 4a or 4b as per the C-TIRADS. Among them, 63 cases were of category 4a nodules, and the pathology findings identified 17 malignant nodules as papillary thyroid carcinomas, the other 46 cases were benign nodules which included 42 nodular goiters and 4 adenomas. The remaining 43 cases out of 106 were of category 4b nodules, and the pathology findings identified 37 malignant nodules which were papillary thyroid carcinomas, the other 6 cases had benign nodules which were nodular goiters.

The conventional US characteristics of 106 thyroid nodules are presented in **Table 1**.

**Table 1:** Conventional US characteristics of 106 category 4a and 4b thyroid nodules.

Characteristics	4a	4b
Nodules(n=106)	63	43
Maximum diameter		
≤1 cm	33	27
1-2 cm	19	12
≥2 cm	11	4
Internal structure		
Solid	61	41
Non solid	2	2
Echogenicity		
Markedly hypoechoic	28	38
Isoechoic or Mixed echoic	35	5
Margin		
ill-defined or irregular	3	35
defined or regular	60	8
Calcification		
Microcalcification	2	4
None or Macrocalcification	61	39
Aspect ratio		
>1	0	6
<1	63	37
Comet-tail artifact		
Present	3	0
None	60	43

The diagnostic efficacy of the C-TIRADS in detecting malignant nodules among category 4a and 4b nodules is presented in **Table 2**.

**Table 2:** Comparison of diagnostic efficacy of three diagnostic methods.

Methods	AUC	Sensitivity (%)	Specificity (%)	Accuracy(%)	PPV (%)	NPV(%)
C-TIRADS	0.785^*^	68.5	88.5	78.3	86.0	73.0
SWE	0.775^**^	68.5	86.5	77.4	84.1	72.6
C-TIRADS+SWE	0.870	83.3^#^	84.6^##^	84.0	84.9	83.0

* indicates the AUC of C-TIRADS compared with that of SWE, z = 0.18, P > 0.05, the AUC of C-TIRADS compared with that of C-TIRADS + SWE, z = 2.76, P < 0.05; ** indicates the AUC of SWE compared with that of C-TIRADS + SWE, z = 2.25, P < 0.05; ^#^ indicates that the comparison of sensitivity values among the three diagnostic methods had statistical significance (P < 0.05); ^##^ indicates that the comparison of specificity values among the three diagnostic methods had no statistical significance (P > 0.05).

### Diagnostic efficacy of SWE in detecting malignant nodules among category 4a and 4b nodules

**Figure 1:**
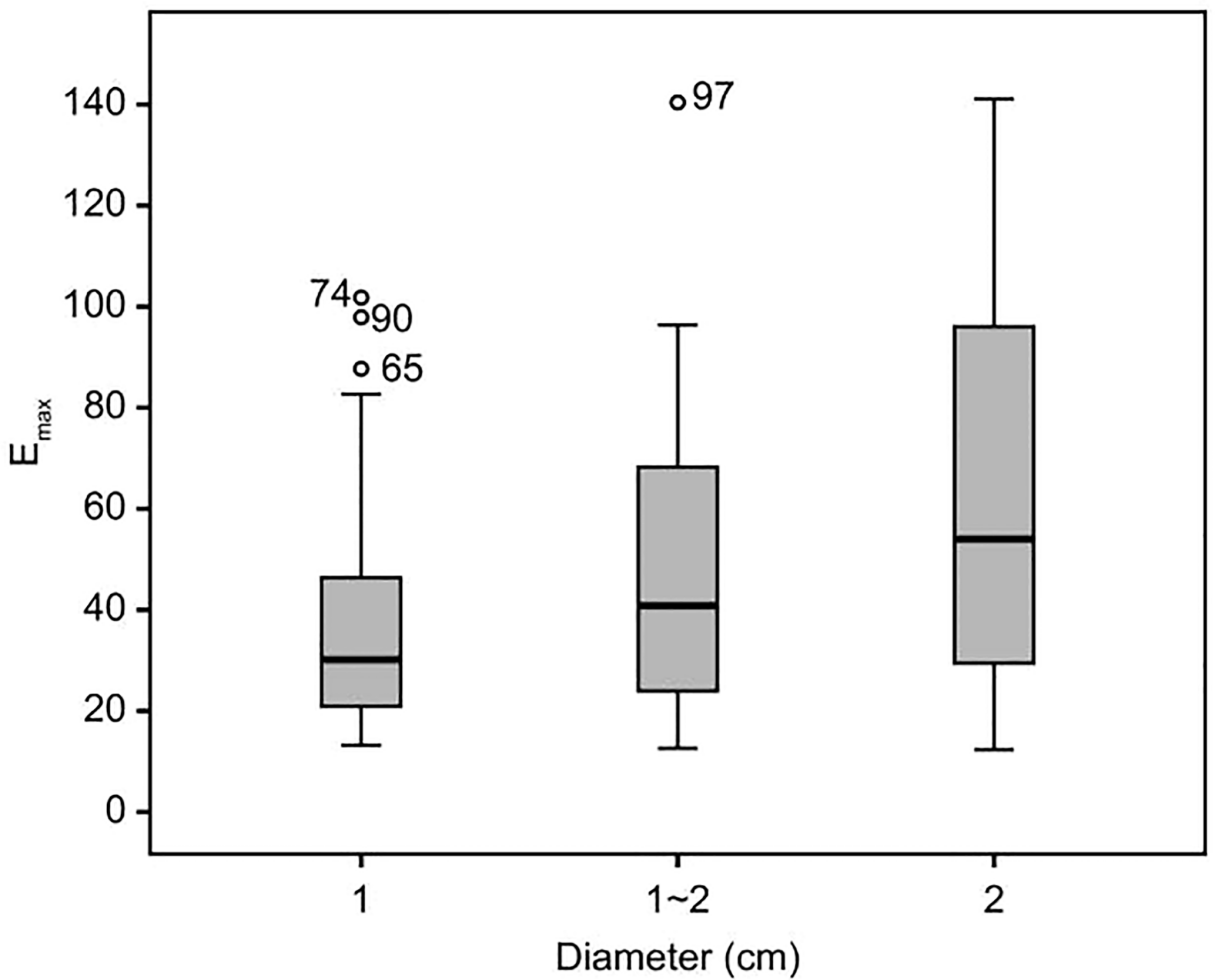
Emax values distribution of 106 category 4a and 4b thyroid nodules. The figure was created using R software (version 3.4.4, url: https://www.R-project.org).

The distribution of Emax values of 106 TIRADS category 4a and 4b nodules is shown in **Figure 1**. The Emax values of the 106 thyroid nodules were non-normally distributed.

Among category 4a nodules, 33 cases had a maximum diameter ≤ 1 cm, 19 cases had a maximum diameter of 1–2 cm, and 11 cases had a maximum diameter ≥ 2 cm. Among category 4b nodules, there were 27 nodules with a maximum diameter ≤ 1 cm, 12 nodules with a maximum diameter of 1–2 cm, and 4 nodules with a maximum diameter ≥ 2 cm.

According to the above Emax diagnostic criteria, 106 cases of benign and malignant nodules were diagnosed. Of the 63 cases of category 4a nodules, 47 were diagnosed as benign, and 16 cases as malignant. Among the category 4b nodules, 30 cases were diagnosed as malignant, and 13 as benign.

The diagnostic efficacy of SWE in detecting malignant nodules among category 4a and 4b thyroid nodules is presented in **Table 2**.

### Diagnostic efficacy of C-TIRADS + SWE in detecting malignant nodules among category 4a and 4b nodules

According to the diagnostic criteria of C-TIRADS + SWE, 16 cases of C-TIRADS category 4a nodules were reclassified to category 4b, and 30 cases of C-TIRADS category 4b nodules were reclassified to category 4C.

The diagnostic efficacy of C-TIRADS+SWE in detecting malignant nodules among category 4a and 4b nodules is presented in **Table 2**.

### Comparison of diagnostic efficacy of the three diagnostic methods

**Figure 2:**
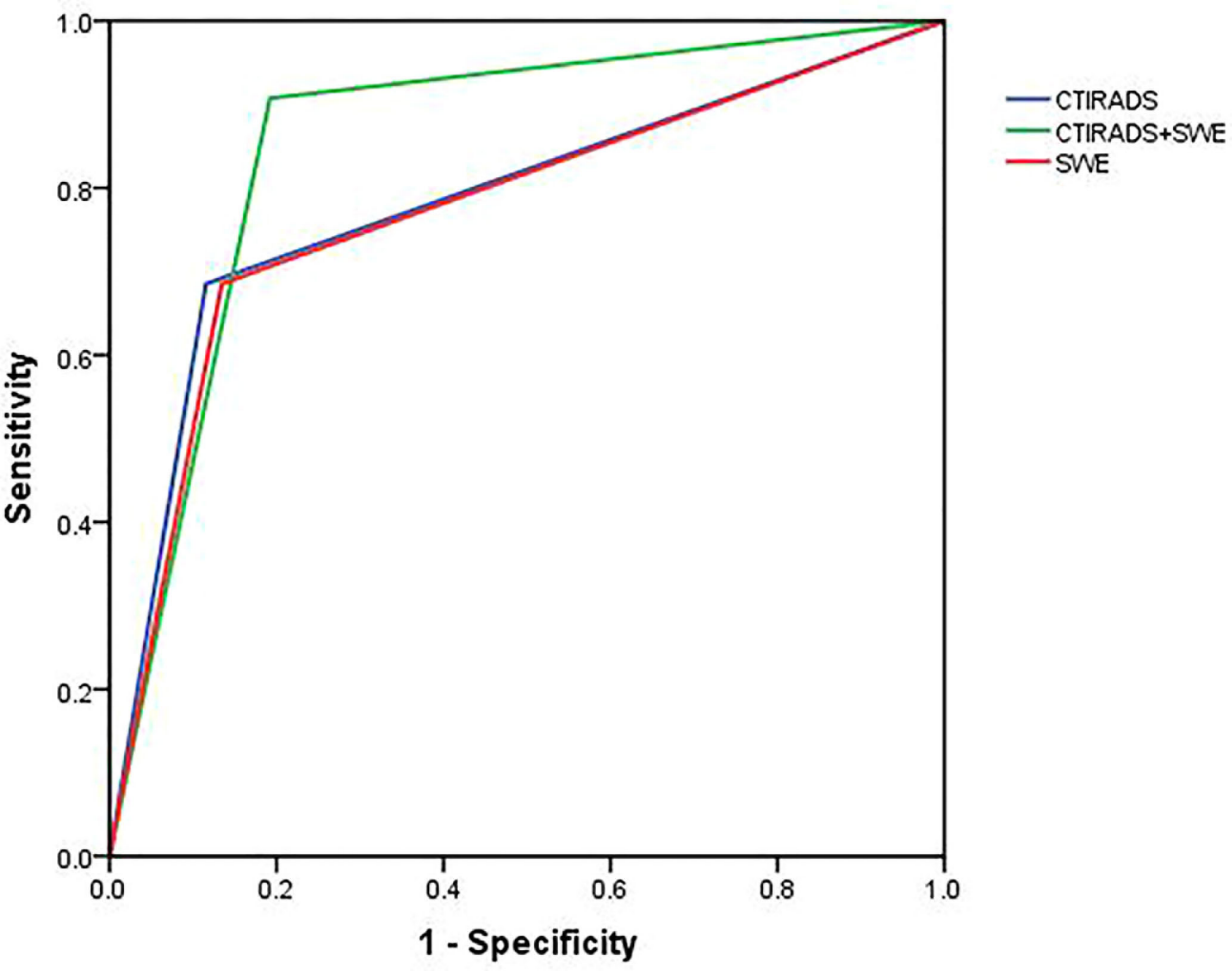
ROC curves to evaluate the efficacy of three diagnostic methods for the diagnosis of category 4a and 4b thyroid nodules. The AUC value of C-TIRADS + SWE was higher significantly compared with that of C-TIRADS (z = 2.76, *P* < 0.05) or SWE (z = 2.25, *P* < 0.05). There was no significant difference in AUC value between C-TIRADS and SWE (z = 0.18, *P* > 0.05). The figure was created using R software (version 3.4.4, url: https://www.R-project.org).

We drew ROC curves to evaluate the efficacy of three methods in the diagnosis of category 4a and 4b nodules (**Figure 2**), while taking the pathology results as the gold standard. The AUC values of C-TIRADS, SWE, and C-TIRADS+SWE in the diagnosis of category 4a and 4b nodules were 0.785, 0.775, and 0.870, respectively. The AUC value of C-TIRADS + SWE was significantly higher compared with that of C-TIRADS (*P* < 0.05) or SWE (*P* < 0.05). The diagnostic efficacy parameters are shown in **Table 2**.

We also compared the sensitivity and specificity values for the diagnosis of category 4a and 4b thyroid nodules among the three diagnostic methods. The sensitivity value of C-TIRADS + SWE (83.3%) was higher than that of C-TIRADS (68.5%) or SWE (68.5%) alone (*P* < 0.05). There was no significant difference among the specificity values of the three diagnostic methods (*P*>0.05).

## Discussion

US is the best imaging method for the thyroid, and plays an important role in the diagnosis and management of thyroid nodules. However, there are often some inconsistencies in terms of the terminology used for reporting, or recommendations for management due to subjective interpretation of the images. In view of this, clinicians across many countries set up the Thyroid Imaging Reporting and Data System (TIRADS) which is specific to thyroid nodules, patterned on the Breast Imaging Reporting and Data System (BIRADS) published by the American College of Radiology (ACR). Since 2009, various versions of TIRADS were successively established (10–18), including the Eu-TIRADS (19) of the European Thyroid Association, and ACR-TIRADS published by the ACR (20). However, the difference in the diagnosis and treatment of thyroid nodules across different countries makes it difficult for many doctors to adopt the risk stratification system of ACR-TIRADS or Eu-TIRADS in their respective countries. Therefore, in this study, we chose the recently released C-TIRADS.

The AUC, sensitivity, specificity, and accuracy of US with C-TIRADS for the diagnosis of malignant nodules among category 4a and 4b nodules were 0.785, 68.5%, 88.5%, and 78.3%, respectively, in this study. Compared with previous studies, the diagnostic efficacy of our results was lower than those of US in the diagnosis of benign and malignant thyroid nodules (7, 21). This can be due to the type of thyroid nodules selected for the investigation. In our study, US was only used to evaluate C-TIRADS category 4a and 4b thyroid nodules and not all thyroid nodules. The malignant features of these nodules were often not obvious, and it was difficult to differentiate between benign and malignant nodules using US alone. In addition, we did not study category 4c thyroid nodules because the malignant features of these nodules were relatively obvious and these were easier to diagnose than category 4a and 4b nodules. However, compared with similar studies that evaluated only category 4 thyroid nodules (3), the diagnostic efficacy of our results was higher, and this may be related to C-TIRADS, the ultrasonic diagnostic standard that we selected for this study.

In our study, there was no significant difference in diagnostic efficacy between SWE and US with the C-TIRADS. The AUC, sensitivity, specificity, and accuracy of SWE in the diagnosis of category 4a and 4b nodules were 0.775, 68.5%, 86.5%, and 77.4%, respectively. Diagnosis of TIRADS category 4a and 4b nodules using SWE had high specificity and low sensitivity, and this was consistent with previous studies (22, 23). In our previous study, we found that the size of thyroid nodules had a great impact on the Emax value of SWE (9). Using different diagnostic cut-off points for different sizes of nodules improved the diagnostic efficacy significantly. Therefore, in this study, we used different cut-off points for different sizes of thyroid nodules when SWE was used to diagnose category 4a and 4b thyroid nodules, and hence, the diagnostic efficacy of SWE was better when compared with other similar studies.

In recent years, there have been many reports on the combination of ultrasound classification systems with elastography or contrast-enhanced ultrasound for the diagnosis of thyroid nodules. Most of them believed that combined methods were helpful for the differential diagnosis of thyroid nodules (5, 24–26). Some studies reported that combining SWE or Virtual Touch Tissue Imaging and Quantifification (VTIQ) with TI-RADS could improve the diagnostic specificity of thyroid nodules (27). Some researches have shown that the modified TI-RADS based on ACR TI-RADS+ SWE+ CEUS could reduce the frequency of FNA for benign nodules and implement consistent follow-up in clinical practice (28). Some studies have shown that the combination of TI-RADS and CEUS could improve the diagnostic accuracy of thyroid nodules, especially for TI-RADS 4 nodules (29). As we found in the present study too, the combined diagnostic method (C-TIRADS + SWE) significantly improved the diagnostic efficacy in detecting malignant nodules among category 4a and 4b nodules, and the AUC, sensitivity, specificity and accuracy were 0.870, 83.3%, 84.6%, and 84.0%, respectively, which might provide a new standard for diagnosis of such nodules. The improvement in diagnostic efficacy effectively reduced the false positive rate and false negative rate, thereby reducing unnecessary fine-needle aspiration (FNA) or surgery.

Our study had some limitations. First, all patients in our study underwent surgery. Therefore, there might be a bias in the selection of this sample which had an increased proportion of malignancy. Second, in this study, the pathological types were relatively singular and most of them were papillary carcinomas and nodular goiters. The diagnostic performance of the above methods for other thyroid pathological types requires further investigation. Last, the sample size in this study was not large enough and further research with larger samples is required.

## Conclusions

In conclusion, in this study, we found that a combination of SWE and US with C-TIRDS was an effective diagnostic method for the differential diagnosis of category 4a and 4b thyroid nodules. While the diagnostic efficacy of these two methods used separately was similar, the combination of SWE and US with the C-TIRADS significantly enhanced the diagnostic efficacy of detecting malignant nodules among category 4a and 4b nodules. This provides a reference for its further use by clinicians in diagnosis and treatment.

## Data availability statement

The original contributions presented in the study are included in the article/supplementary material. Further inquiries can be directed to the corresponding author.

## Ethics statement

The studies involving human participants were reviewed and approved by Shanxi Bethune Hospital. The patients/participants provided their written informed consent to participate in this study.

## Author contributions

Conception and design of the research: CK and HL; Acquisition of data: JX and YZ; Analysis and interpretation of the data: YZ and JM; Statistical analysis: LJ and JM; Obtaining financing: JX; Writing of the manuscript: HL; Critical revision of the manuscript for intellectual content: CK, HL, and LJ. All authors contributed to the article and approved the submitted version.
